# Multidisciplinary team decision-making in cancer and the absent patient: a qualitative study

**DOI:** 10.1136/bmjopen-2016-012559

**Published:** 2016-07-21

**Authors:** D W Hamilton, B Heaven, R G Thomson, J A Wilson, C Exley

**Affiliations:** 1 Institute of Health and Society, Newcastle University, Newcastle upon Tyne, UK; 2 Head and Neck Surgery, Freeman Hospital, Newcastle upon Tyne, UK

**Keywords:** ORAL & MAXILLOFACIAL SURGERY

## Abstract

**Objective:**

To critically examine the process of multidisciplinary team (MDT) decision-making with a particular focus on patient involvement.

**Design:**

Ethnographic study using direct non-participant observation of 35 MDT meetings and 37 MDT clinics, informal interviews and formal, semistructured interviews with 20 patients and 9 MDT staff members.

**Setting:**

Three head and neck cancer centres in the north of England.

**Participants:**

Patients with a diagnosis of new or recurrent head and neck cancer and staff members who attend the head and neck cancer MDT.

**Results:**

Individual members of the MDT often have a clear view of which treatment they consider to be ‘best’ in any clinical situation. When disagreement occurs, the MDT has to manage how it presents this difference of opinion to the patient. First, this is because the MDT members recognise that the clinician selected to present the treatment choice to the patient may ‘frame’ their description of the treatment options to fit their own view of best. Second, many MDT members feel that any disagreement and difference of opinion in the MDT meeting should be concealed from the patient. This leads to much of the work of decision-making occurring in the MDT meeting, thus excluding the patient. MDT members seek to counteract this by introducing increasing amounts of information about the patient into the MDT meeting, thus creating an ‘evidential patient’. Often, only highly selected or very limited information of this type can be available or known and it can easily be selectively reported in order to steer the discussion in a particular direction.

**Conclusions:**

The process of MDT decision-making presents significant barriers to effective patient involvement. If patients are to be effectively involved in cancer decision-making, the process of MDT decision-making needs substantial review.

**Strengths and limitations of this study**

- This study is an in-depth ethnographic study of team decision-making in head and neck cancer and our findings may be applicable to other circumstances where team decision-making is used (in cancer or other disease states).
- It is the first study to analyse transcribed audio-recorded data from team meetings and clinic appointments and provides a novel insight into the challenges facing MDTs when involving patients in decisions.
- Data were collected from only three centres in the north of England and as such do not represent a broad view of multiple team and clinic manifestations which may be present in other centres.
- Some of the challenges described may have been tackled in other departments by variations in the structure of the team meeting and clinic.

## Introduction

Multidisciplinary team (MDT) working and team decision-making is currently the standard of care in cancer treatment decision-making and all patients with cancer should have their treatment plan discussed in an MDT.^1^ The MDT was introduced after the ‘Calman-Hine report’^2^ that was written as a response to unaddressed variation in care across the UK^3^ ^4^ and an increasing weight of evidence suggesting that those patients treated in specialist cancer centres had better outcomes than those who were not.^5^ ^6^ MDT working is thought to improve cancer staging accuracy,^7^ recruitment to clinical trials,^8^ ^9^ adherence to quality-of-care indicators,^10^ patient satisfaction and time to treatment.^11^ However, the introduction of MDT working has not resulted in a demonstrable improvement in cancer survival.^12^ Indeed, although a treatment plan devised by an MDT often differs from that of a single clinician,^13–15^ whether or not it is a superior decision is unclear. MDTs are also resource-intensive: the meeting and the time taken to prepare for it are time consuming.^16^ The estimated cost of all UK MDT meetings is £50 million a year for preparation and the same amount again for attendance.^17^

It is ethically important to make good, individualised healthcare decisions that respond effectively to the needs of the patient. Using decision aids to help share decisions with patients results in improved patient knowledge, lower decisional conflict, increased patient satisfaction with the decision-making encounter and improved perception of risk.^18^ Well-informed patients make different decisions to those who are less well informed,^19^ may perceive risk in a different way^20^ and adhere better to treatment.^21^ Doctors who do not effectively involve patients in decisions about their health may feel that they are working in the best interests of the patient, but in fact may not arrive at a decision that is right for that particular patient. When the treatment priorities of patients and clinicians are compared in head and neck cancer (HNC), they are invariably found not to match^22–26^ and this finding is echoed in other cancers.^27^ ^28^ If the aim of the MDT meeting and clinic is to make appropriate individualised treatment decisions, then the values and preferences of the patient should be central to the process. However, there is emerging evidence that the process of MDT working presents barriers to effective involvement of the patient in decision-making.^29^ ^30^ MDTs often struggle with how and when to incorporate individual patient information such as treatment preference into the discussion.^31^ ^32^

Patients and clinicians face particular difficulties with decision-making in HNC. Almost a third of patients with HNC will die of their disease within 2 years, while treatment may cause persistent and debilitating effects on swallow, voice and quality of life. In order to achieve cure of the disease, patients often need to trade-off swallow or voice function; hence, treatment decision-making in HNC is an excellent context for exploring MDT decision-making. This article aims to critically examine the process of MDT decision-making with a particular focus on how the MDT decision-making process involves patients.

## Methods

**Figure 1:**
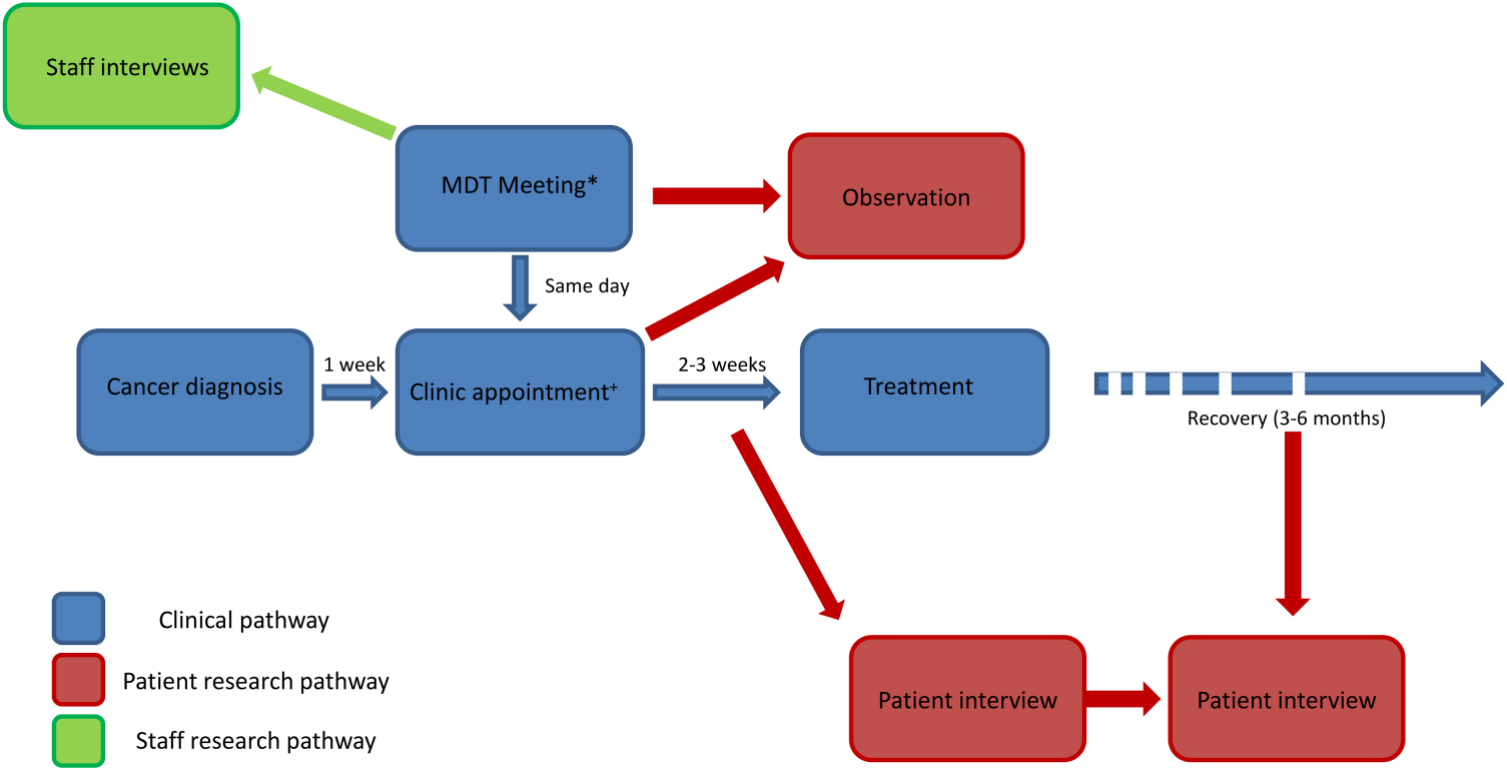
Study flow chart. *MDT meeting between staff members: patient is discussed, imaging and pathology reviewed, but patient is not present. ^+^Clinic appointment between some members of the MDT and the patient, where treatment decision is made. MDT, multidisciplinary team.

This qualitative study used non-participant observation and semistructured interviews to critically examine how decisions were made in the MDT (see figure 1). All data were collected by one researcher (DWH). Non-participant observation enables the researcher to study participants in their natural environment, and adds value to retrospective accounts gleaned only through participant interviews which may provide idealised accounts of events.^33^

### Ethical approval

Ethical approval was gained from the NHS Research Ethics Newcastle and North Tyneside 2 committee (reference 11/NE/0200) in September 2011. Prior to the commencement of data collection in each centre, all necessary local Research and Development governance permissions were obtained.

Before interviews, written consent was obtained from participants; however, for observations, a lengthy process of informing potential participants about the study was followed (with opportunity for questions and cooling off) and then verbal consent.

#### Observations

The research was conducted in three HNC centres in the north east of England. Non-participant observations of 35 MDT meetings and 37 MDT clinics were conducted, and from these, 30 patients with HNC were sampled (table 1). Patients were excluded from the study if they did not understand written or spoken English or they did not have the capacity to consent. The MDT meetings and clinics were all audio-recorded and transcribed verbatim. Detailed field notes were also made at the time of observation, then transcribed immediately afterwards.

**Table 1:** Details of included participants and data collection

				Observation			
Patients: group 1	Centre	Age	Tumour site	MDT	Clinic	Int 1	Int 2
James Cain	A	68	Pharynx	1	1	1	1
Frances Cotton	A	82	Pharynx	1	1	x	x
Philip Vase	A	61	Parotid	1	1	x	x
Fred Barnes	A	71	Lip	1	1	x	x
Deborah Dolphin	A	54	Pharynx	1	1	1	x
Vincent Lowry	A	80	Pharynx	1	1	x	x
David Forcett	A	72	Pinna	1	1	x	x
Stanley Wright	A	87	Pharynx	1	1	1	x
Daniel Carding	A	64	Larynx	1	1	1	x
John Winton	A	61	Larynx	1	1	1	x
Bobby Older	A	52	Pharynx	1	1	x	x
Samuel Black	A	55	Pharynx	1	1	1	1
Keith Down	A	62	Larynx	1	1	1	x
William Runman	B	73	Pharynx	3	1	1	x
Andrew Driver	B	49	Pharynx	1	1	1	x
Donna Childs	B	52	Pharynx	1	1	1	x
David Jobling	B	63	Larynx	1	1	x	x
Sophie Leicester	B	49	Larynx	1	1	x	x
Edward Doman	B	73	Mouth	3	1	1	x
Eric Francais	B	65	Larynx	1	1	1	x
Gary Duck	B	57	Pharynx	1	1	x	x
Jean Dixon	B	63	Pharynx	1	2	1	1
Jane Doe	C	69	Pharynx	1	2	1	x
Margaret Brigstock	C	81	Mandible	1	2	x	x
Roy Dayson	C	60	Pharynx	1	1	1	x
Dana O'Malley	C	67	Pharynx	1	1	x	x
Gary Nicholson	C	46	Pharynx	1	2	x	x
Tracey Burnham	C	38	Larynx	1	1	x	x
James Matfield	C	70	Larynx	1	4	1	x
David Dale	C	84	Larynx	1	1	x	x
Kevin Hair	A	82	Pharynx	x	x	1	x
David Newman	A	57	Larynx	x	x	1	x
Frank Sunnyman	A	52	Pharynx	x	x	1	x
Phil Gardener	B	65	Larynx	x	x	1	x
**Staff (interview only)**	**Centre**	**Role**					
Mr Red	A	ENT surgeon					
Dr Brown	A	Oncologist					
Mr Surton	A	Maxillofacial surgeon					
Mrs Pope	A	Speech and Language Therapist					
Tessa Dark	A	Clinical Nurse Specialist					
Mr Halifax	B	Maxillofacial surgeon					
Mr Blaydon	B	ENT surgeon					
Mr North	B	ENT surgeon					
Dr Goodier	C	Oncologist					

Int, interview; MDT, multidisciplinary team.

### Interviews

Semistructured interviews were conducted with patients and staff (see table 1). In a semistructured interview, a researcher has a topic guide with the topics of discussion or specific questions,^34^ but the format allows the participant to talk in more depth on certain subjects. This means that the resultant data are comparable, but rich and in depth. The development of the interview guide was iterative; as data collection continued, the content of the guide evolved in order to explore emerging themes. In total, 20 patients were interviewed between the treatment decision and the commencement of treatment. Four of these patients were interviewed again 9 months following the completion of treatment. Semistructured interviews were also conducted with nine MDT members (five surgeons, two oncologists, one speech and language therapist and one clinical nurse specialist; see table 1). In addition to these formal interviews, through the course of the observations, informal interviews with staff members of the MDT also took place which, although not audio-recorded, were incorporated into written field notes. Pseudonyms were used for reporting data throughout to protect the anonymity of respondents.

### Sampling

Purposive sampling was used throughout the study in order to build concepts and ask questions of the emerging data. Data collection and analysis occurred in tandem, and further sampling was guided by the emerging analysis.^35^ Patients and staff members were sampled with the aim of exploring, developing or challenging the emerging concepts and themes from the prior analysis. Sampling continued until a state of theoretical sufficiency^36^ was achieved.

### Analysis

The data were analysed by one researcher (DWH) following principles of constructivist grounded theory.^37^ First, line-by-line open coding was performed on a small number of transcripts and from this, an initial coding framework was produced. Following this, all transcripts were coded using axial coding, based on the initial coding framework. The emerging analysis was used to guide further sampling and further development of the coding framework; when the coding framework was altered, all transcripts were recoded. The emerging coding framework and analysis was discussed in depth with BH and CE at regular intervals, and with the wider research team. In line with constructivist grounded theory, the codes used were conceptual, rather than descriptive, and labels were derived completely from the data, not predetermined. The coding was organised using the NVivo version 9 computer package. Memos were used to develop a deeper description of the codes and the data, and allowed the development of theory which again was used to guide further sampling.

## Results

In all centres studied, the MDT meeting was attended by a range of medical specialities and allied healthcare professionals, and took place before the patient was seen in the MDT clinic. The patient was not present at the MDT meeting; hence, it was a ‘backstage’ area^38^ where staff members could talk more openly about the patient, their treatment options and their prognosis. The aim of the discussion was to reach a consensus on which treatment or treatments were considered to be the ‘best’; this treatment recommendation was then delivered to the patient in the MDT clinic, a ‘frontstage’ area where the patient is present (see figure 1). Many individual clinicians had their own strongly held view of what they feel is ‘best’ in certain clinical situations:

In general head and neck there often isn't an option; you've got the best treatment that there's an evidence base for that…once you've decided on that option…that is the truth as far as we know it…the patient's decision…may not be ‘I want radiotherapy, I want surgery’, it's do you want the best treatment we know of or not.

(Dr Brown, Oncologist, Interview)

Often, the members of the MDT agreed that one option was clearly the ‘best’ treatment for a particular patient. If this was the case, the option was usually delivered by a member of the MDT as a single recommendation for treatment to the patient in the MDT clinic. However, at other times, such agreement did not occur; rather, different clinicians held different views about what was the ‘best’ option. In this situation, although all options were presented to the patient by a clinician, the clinician's description of the treatment options was often ‘framed’. Framing is where the options are not presented in a balanced way, but with an emphasis that makes it difficult for the patient to do anything other than comply with the speaker's assessment of ‘best’ or at least tend towards favouring one option over another. The following data extract is an oncologist's description of radiotherapy given to a patient in clinic:

Dr Green: It's a very accurate treatment. …You don't feel anything. You just lie there and then you go home again. …But, the radiotherapy does cause some side effects and they can be quite nasty. Obviously the aim of the radiotherapy is to try and get rid of this cancer and to do that we have to give quite big doses of the radiotherapy. …So your skin on the outside will start getting red like it's had a sun burn-type reaction and on the inside it starts getting red and inflamed as well. And that means that you'll start having problems like a sore throat and some problems with your swallowing. …And that means that you'll need lots of support as you go through the treatment.

(David Dale, Observation, MDT Clinic)

Here the emphasis on the positive aspects of the treatment can be seen; comparisons were made to previous experiences that are undoubtedly unpleasant and difficult, but certainly not overpowering. The next extract is to a similar patient in a different clinic; the oncologist was explaining the process and side effects of radiotherapy:

Dr Goodier: We need to spread it out over six weeks of daily treatment. That means you coming up from home, Monday to Friday, every day for six weeks with just gaps at the weekend. …We would lie you on a couch on your back, wide awake. …As the treatment goes through, your body starts reacting to the radiation that we're giving it. …Everything becomes inflamed and sore. The outside of your skin and the inside of your throat will all become quite red and hot and sore and that's why swallowing will become very, very difficult—probably impossible. Even swallowing your own saliva will be impossible by the time you get to the end of that six weeks.

(Gary Nicholson, Observation, MDT Clinic)

In both of these extracts, the patient had a choice between treatment options. The comparison of these data extracts demonstrates how the description of a treatment option could have a fundamental effect over the treatment choice made by the patient. It would be understandable for the patient to choose the first description, and turn down the second. This means a doctor can offer choice (in that two options can be described and offered), without offering properly informed choice.

### Presentation of treatment options

The MDT members regularly disagreed on which treatment was best or felt that two or more treatment options could equally be considered for an individual patient. A difference of opinion may be due to varying interpretations of the clinical evidence or the research literature, or alternative conceptualisations of how the ‘best’ treatment can be defined. The following extract is from the MDT meeting discussion about Samuel Belton, a 55-year-old man with a moderately sized (T2) cancer of the tonsil. At this point in the discussion, some members of the team felt that the patient should have a major resection and reconstruction, whereas others felt that he should have radical chemoradiotherapy:

Mr Surton [Maxillofacial surgeon]: So shall we see him together

(Silence 10s)

Mr Jones [ENT surgeon]: Depends how you put it to the patient isn't it, you know!

Mr West [Plastic surgeon]: It's one of these things, we've done this before, and you see the patient, and you have two people there, and you confuse the patient even more. I think…

Tessa [Clinical Nurse Specialist]: It's horrendous, I think it is the worst thing you can do for a patient

Mr West: I agree. I think it's a terrible thing

Tessa: Patients just don't know, they just don't know what to do

Mr Surton: But are we not supposed to give the patient choice?

Mr West: And if we can't decide, I think it's really unfair, I know we've been here before and we've spoken about it, but this is…

Tessa: Hey I've seen…, you know it's picking up the pieces afterwards, because they really cannot make that decision.

(Samuel Belton, Observation, MDT Meeting, Centre A, 1 March 2012)

Importantly, this particular discussion did not centre on the merits of the particular treatments: the individual members had interpreted the available evidence and come to different conclusions. Instead, the discussion was about whether the MDT members should come to a consensus in the MDT meeting or offer the options for treatment to the patient.

In the discussion above, the MDT as a group had two treatment options available for the patient. However, individuals within the group disagreed on which option was better. This led to difficulty in deciding who would communicate the final view of the MDT to the patient. Different clinicians may frame their description to their own individual view of which is ‘best’, as previously shown. In the extract above, this was acknowledged with the comment from Mr Jones, ‘Depends how you put it to the patient isn't it, you know!’. MDT members are aware of the effect of framing on the delivery of the treatment recommendation(s) in the MDT clinic. The following data are taken from observation of an MDT meeting for Philip Vase discussing whether he should be offered postoperative radiotherapy or not:

Dr Brown (oncologist): I'd give him radiotherapy but…would you keep him under observation then?

Dr Yellow (oncologist): [nods]

Dr Brown: I think you would cause not really very much morbidity [with radiotherapy], it's so peripheral and so lateral. So…if you want him treated, send him to me, if you want him under observation, send him to Dr Yellow.

(Philip Vase, Observation, MDT meeting)

These data clearly demonstrate that the MDT members acknowledged that framing exists. Thus, the choice of who delivers the recommendation can become a proxy for the recommendation itself. This not only demonstrates the difficulties the MDT face when there is more than one option but arguably undermines the whole MDT decision process.

### Uncertainty

The difference of opinion demonstrated by the case of Samuel Belton above was seen by many members of the MDT as something which should remain in the backstage MDT meeting, and not be presented to the patient.

I really do not believe it's fair to say to the patient, “We're quite uncertain, we don't know what to do, these are the options, what do you want?” I mean that is just [terrible] because you're the…expert, that's what they're paying you for, “What would you do?” and you have to be able to say, “I would—if this was my mum, that's what I'd do.” I think they should leave with a degree of certainty about their treatment. …They can't walk away thinking, “…even the experts don't know what to do.” That's desperately wrong.

(Mr Halifax, Maxillofacial Surgeon, Interview)

In this extract, Mr Halifax linked the provision of certainty to his status as a professional or expert. Uncertainty, on the other hand, was seen as not knowing what to do: he saw one of the roles of the MDT meeting as creating certainty through a clear recommendation. Thus, although the MDT is designed to bring the various opinions of the expert members together in a backstage setting, when these opinions are not aligned with one another, the group faces difficulty and disagreement on how to proceed. Here, Mr Halifax views offering choice as potentially abandoning the patient (‘these are the options, what do you want’), which then acts a barrier to patient involvement.

### The ‘evidential patient’

The desire of some MDT members to conceal uncertainty and difference of opinion from the patient led to a lot of the work associated with decision-making occurring in the backstage MDT meeting, excluding the patient. The patient is absent from the MDT discussion and MDTs counteract this by creating an ‘evidential patient’ in the backstage meeting using information about them. The evidential patient was almost universally based on clinical information about the cancer size, extent and spread; this was usually presented first and thus forms the basis of the discussion. However, team members recognised that this clinical information alone did not provide a complete view of the patient:

You're just making decisions based on scans and guidelines as opposed to an individual ever having had the opportunity to explore not just their physical and psychological status, but their feelings about treatment…it would be nice to have much more of a feel and a knowledge of the patient before it gets to that MDT.

(Mrs Pope Speech and Language Therapist, Interview)

Mrs Pope expresses the difficulty in representing a holistic view of a patient in a backstage setting. Her statements reveal that the information provided often does not include the patient's lifestyle, context, values or preferences. When information other than clinical details of the tumour was available about the patient (comorbidities, social situation, support network or even stated values or preferences), this could have a significant guiding effect on the discussion:

Mr Jones (ENT surgeon): He's a very sort of straightforward sort of man, who doesn't worry too much, but he will probably cope with [the diagnosis] very well. But, he needs a lot of radiotherapy

Dr Brown: What age is he?

Mr Jones: He's 87, I mean he's a very good 87.

(Stanley Wight, Observation, MDT meeting)

In this extract, it appears that there was a judgement made by Mr Jones about the patient being a ‘very good 87’ years old. On one level, this provided more information about the patient, and helped to form a picture of the person that he is. On another level, it is a judgement that is intended to sway the team in one direction, towards more radical management (this is what the team recommended to the patient). These small but important details often had vital guiding effects on the tone of the discussion, which pushed the direction of the MDT decision-making towards or away from specific treatment options.

## Conclusion

This detailed study is the first to explore the entire MDT meeting and clinic decision process and has shown that MDT decision-making presents significant barriers to engaging with patient values and preferences, and thus presents obstacles to delivering shared decision-making. Although many members of the MDT have a clear view of which treatment they consider to be ‘best’ in a given clinical situation, they are not always in agreement with one another. Any discussion of which treatment is ‘best’ depends on the values of the individual, and thus any disagreements may reflect the differing values of the MDT members. This only reinforces the argument for making the values and preferences of the patient central to the decision process. When team members disagree, the MDT face difficulty in presenting this to the patient. First, many MDT members feel that any disagreement and difference of opinion in the MDT meeting should be concealed from the patient. Second, MDT members recognise that the clinician selected to present the treatment choice to the patient may ‘frame’ their description of the treatment options to fit their own view of best. This makes the choice of clinician to deliver the treatment choice a proxy for the recommendation itself. Third, many MDT members see presenting treatment options to the patient as abandoning them without guidance from the MDT. This combination leads to a lot of the work of decision-making (negotiating risks, trading off function and survival, etc) occurring in the MDT meeting, thus excluding the patient. Although MDT members attempt to counteract this by introducing clinical and non-clinical information about the patient into the meeting (the ‘evidential patient’), this cannot be construed as greater involvement of the patient in decisions. The resultant recommendation, although perceived as more ‘patient-centred’, cannot adequately include the values and preferences of the patient and thus does not constitute involvement of the patient.

Information about the social situation, character, values or preferences of the patient is central to a personalised treatment decision. Information of this nature holds particular power within the meeting, with a clinician who has met and spoken to the patient holding ‘encountered authority’ over the decision-making process.^39^ However, often only highly selected or very limited information of this type can be available or known and it can easily be selectively reported in order to bias or steer the discussion. Also, any attempt to include information such as a patient preference as a stable information commodity which can be passed from person to person in the MDT meeting is problematic. Patient preferences that are formed in life-threatening situations are unlikely to be preformed or pre-existing^40^ and as a result are often unstable.^41^ Patients' expressed preferences are constructed during elicitation^40^ ^42^ and thus are labile, dynamic, reversible and sensitive to option description: hence, they are the product of an interaction.^43^ Furthermore, if the patients' preferences are indeed elicited, they may at first be uninformed and taken at face value rather than explored and discussed. In the current structure, the MDT faces difficulty in moving from these ‘initial preferences’ to ‘informed preferences’^44^ as this cannot be performed in the absence of the patient.

The MDT cannot make decisions that involve a patient by introducing increasing amounts of information about the patient into the backstage MDT meeting. Instead, the MDT decision process should be structured to maximise the interaction between the members of the team and the patient. Also, the current model of MDT decision-making gives ample opportunity for clinicians to discuss and negotiate aspects of the decision with each other but does not afford patients the same opportunity. When patients' processes of decision-making have been explored, they have been found to be distributed among people, places and information sources.^45^ Patients take the information which they have available to them and share it with those important to them, trading off and negotiating. Indeed, in this way, patients behave in much the same way as the MDT members at the MDT meeting.

This study represents the first ethnography of the MDT meeting and the clinic combined and provides a detailed analysis of the process of decision-making in this setting. However, MDT structure and practice will vary geographically, by disease and by patient group. Although the MDT settings studied here may not precisely reflect the particular MDT set-up in other centres, the concepts and challenges described may be applicable to other situations in which team decision-making is used, especially in cancer care. Quantitative measures of the MDT discussion have measured the ‘patient-centredness’ of the backstage discussion by counting the number of times patient information (demographics, comorbidities, supportive needs, etc) are mentioned in the MDT meeting. Not only has this often been found lacking,^46^ ^47^ but MDTs that include more information about the patient in their meeting cannot be considered to make ‘patient-centred’ recommendations. Previous qualitative studies have described the predominance of the biomedical model of disease in the MDT discussion and the difficulties that MDTs face in not only representing the values or preferences of a patient in the backstage but also incorporating them in a meaningful way into the discussion.^31^ ^32^

Sharing decisions with patients is of central importance to good quality, safe healthcare delivery.^48^ Although the introduction of the MDT has increased the opportunity for professionals to be included in cancer treatment decisions, opportunity for the patient to be involved has been diminished. Many of the initial proposed benefits of the MDT as proposed in the Calman-Hine report may have been realised, but the significant shift to recognising the importance of patient involvement in decision-making seriously questions the sustainability of the current model. Our results emphasise the major limitations inherent in the current mode of team decision-making. If patients are to be effectively involved in decision-making, there is a need for a substantial review of the approach in light of the recognition of the centrality of patients in decisions about their own treatment. Now is the time to propose and test different models of decision-making for cancer (and other MDT-based decisions), which retains the benefits of an MDT approach but place the patient at the centre of decision-making.
